# Sex-specific influence of Lipoprotein(a) levels on coronary plaque characteristics: - The COPRODUCTION Registry -

**DOI:** 10.1007/s00392-025-02770-w

**Published:** 2025-10-09

**Authors:** Philipp Breitbart, Christoph Liebetrau, Dimitri Grün, Holger Eggebrecht, Edelgard Lindhoff-Last, Dirk Westermann, Thomas Voigtländer, Axel Schmermund

**Affiliations:** 1 Department of Cardiology and Angiology, Medical Center – University of Freiburg, Faculty of Medicine, University of Freiburg, Südring 15, Bad Krozingen, 79189 Germany; 2 Cardioangiological Center Bethanien (CCB), Frankfurt, Im Prüfling 23, 60389 Germany; 3 AGAPLESION Bethanien Hospital, Frankfurt, Im Prüfling 21-25, 60389 Germany

**Keywords:** Cardiac imaging, Coronary computed tomography angiography, Coronary artery disease, Coronary plaques, Lipoprotein (a)

## Abstract

**Background:**

Elevated Lipoprotein(a) Lp(a) levels are associated with coronary atherosclerosis as detected by cardiac computed tomography angiography (CCTA). However, quantitative data including coronary plaque volumes and characteristics are scarce. The current study evaluated the sex-specific correlations between (Lp(a)) levels and the extent and composition of coronary stenosis and plaques.

**Methods:**

1,946 patients undergoing CCTA (third-generation dual-source scanner) for suspected coronary artery disease were included whose Lp(a) levels were available. Lp(a) values ≥ 125 nmol/L were classified as high.

**Results:**

High Lp(a) levels were observed in 336 patients, who had greater maximum degree of stenosis (49.5 ± 26.4% vs. 43.5 ± 27.6%, *P* = 0.002), mainly as a result of the pronounced difference in males (53.8 ± 26.0% vs. 46.2 ± 26.8%, *P* = 0.001). A strong correlation between higher Lp(a) values and high-risk plaque features was noted in the overall cohort (odds ratio [OR]: 1.645; 95% confidence interval [CI]: 1.011 to 2.593; *P* = 0.037), independent of age and LDL-cholesterol values. In males, high Lp(a) levels were associated with greater total plaque volumes (118.1 [IQR 18.3–284.4] vs. 83.2 [IQR 11.8–226.3] mm^3^, *P* = 0.018, Pint = 0.09), including fibrotic components (27.6 [IQR 2.1–109.9] vs. 18.2 [IQR 0.4–65.0] mm^3^, *P* = 0.011, Pint = 0.013), whereas in women, only a marginal linear correlation with total plaque volume was observed (19.2 vs. 8.1 mm^3^; Spearman’s rank correlation R = 0.16, *P* = 0.037).

**Conclusions:**

Our study identifies novel sex-specific correlations between Lp(a) levels and coronary plaque characteristics. High Lp(a) levels in men seems to be associated with increased fibrotic plaque volumes and may contribute to greater total plaque burden and high-risk plaque features.

**Graphical Abstract:**

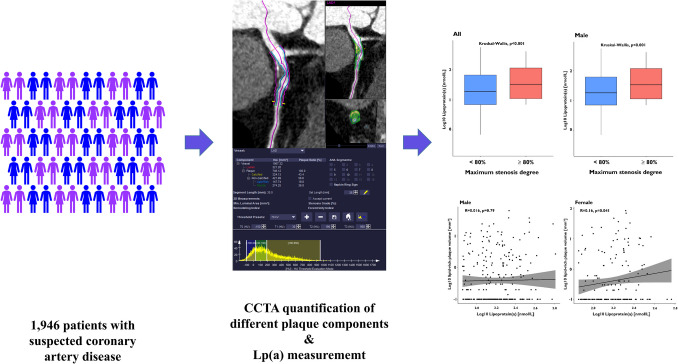

**Supplementary information:**

The online version contains supplementary material available at 10.1007/s00392-025-02770-w.

## Introduction

Lipoprotein(a) [Lp(a)] is a well-established risk factor for coronary artery disease (CAD) [1].

Extensive multiethnic studies from the United Kingdom and United States have established that the risk associated with Lp(a) levels increases linearly, with consistent risks observed across different populations [2, 3]. Consequently, the American National Lipid Association and the European Atherosclerosis Society recommends Lp(a) testing at least once in a person’s lifetime, assigning it a class I rating [4, 5].

Previous studies have demonstrated a significant association between elevated Lp(a) levels and subclinical atherosclerosis, as detected by coronary computed tomography angiography (CCTA). This association applies to any coronary plaques, including both calcified (coronary artery calcium, CAC) and non-calcified subtypes. However, so far, no differentiation of non-calcified plaque composition or focus on high-risk features has been presented [6, 7]. This is important, because the presence of high-risk plaque features, such as low attenuation plaques (LAP), positive remodeling, or high-grade stenoses, is known to be associated with an increased incidence of major cardiac events [8, 9].

As opposed to invasive coronary angiography, CCTA-derived parameters include inherently 3-dimensional and quantitative information on total and non-calcified plaque volumes, including non-obstructive atherosclerosis and high-risk plaque features that can predict future coronary events [9]. A smaller study involving 191 patients with stable CAD demonstrated a significant correlation between high Lp(a) levels (≥ 70 mg/dL) and the progression of LAP over one year, as detected by CCTA [10].

A recent study in patients undergoing invasive angiography revealed that elevated Lp(a) levels are associated with more severe presentations of CAD, including chronic total occlusion (CTO) lesions and diffusely narrowed vessels [11]. Currently, comprehensive data on the correlation between Lp(a) levels and coronary plaque burden, plaque composition, the incidence of significant stenosis, and their impact on clinical outcomes are lacking.

Here, we investigate the baseline correlations between Lp(a) values and extent, severity, and composition of coronary plaque burden in a large outpatient cohort undergoing CCTA for suspected CAD. It was our aim to analyze the influence of Lp(a) levels on coronary plaque burden and high-risk, with a focus on potential sex-specific differences in this association.

## Methods

### Study population

This analysis of the COPRODUCTION “Comparison of prognostic utility and additive value of quantitative coronary plaque volumes assessed with coronary computed tomography angiography and quantitative myocardial ischemia assessed by cardiac magnetic resonance imaging” registry includes all consecutive patients who underwent CCTA for clinical indications and had Lp(a) measurements between July 2017 and June 2020. July 2017 was chosen as the starting point because the same consistent and standardized assay method for Lp(a) measurement was implemented at that time and has been used without change since.

The entire COPRODUCTION registry aims to include approximately 10,000 patients who underwent CCTA for clinical indications at the Cardioangiological Center Bethanien (CCB; Frankfurt/Main, Germany), all scanned using the same CT system since August 2014. Lp(a) measurements have been routinely performed in all patients seen in our in-house outpatient cardiology practice, as part of cardiovascular risk assessment.

Further inclusion criteria were age > 18 and written informed consent for the anonymized use of clinical, procedural, and follow-up data. Exclusion criteria were 1) acute myocardial infarction, 2) CTO, 3) previous coronary intervention and/or coronary artery bypass grafting, 4) incomplete CCTA examination of the coronary arteries (e.g. no contrast medium given due to high Agatston-Score, renal insufficiency, or allergy) or insufficient CCTA image quality or 5) co-morbidities with life-expectancy < two years, and 6) participation in another clinical trial.

This study was approved by the local institutional review board (ethics committee of the Landesärztekammer Hessen, IRB number 2021-2543_1-evBO) and complies with the Declaration of Helsinki. The analysis was funded by Novartis Pharma GmbH. Siemens Healthineers provided the plaque analysis software. Novartis Pharma GmbH and Siemens Healthineers had no influence on the study conception as well as data collection, analysis and interpretation.

### Diagnostic procedures

All patients received a routine cardiac examination including medical history, physical examination, electrocardiogram (ECG), and transthoracic echocardiography. Potential symptoms as well as current medication were recorded. Cardiovascular risk factors were documented, comprising smoking history, history of diabetes and/or hypertension, and hypercholesterolemia. Laboratory measurements included high-sensitivity troponin T (hsTnT), creatine kinase (CK), and N-terminal pro-b-type natriuretic peptid (NT-proBNP), as well as an extensive lipid status (HDL and LDL cholesterol, triglycerides, Lp(a)). Lp(a) was measured by using a fully automated particle-enhanced turbodimetric immunoassay on a Cobas c 303 analysis system (Roche Diagnostics), high Lp(a) levels are defined as values ≥ 125 nmol/L[5, 12]. Cholesterol values were analyzed by using immunoseparation and a fully enzymatic assay (Roche Diagnostics).

### Computed tomography image acquisition

All CCTA examinations were conducted using the same third-generation dual-source scanner, belonging to the newest technique generation for most of the study period (Siemens SOMATOM Force, Siemens Healthineers, Erlangen). Our CCTA protocol has been published[13]. Briefly, we initiated a non-contrast-enhanced scan to calculate the Agatston calcium score prior to administering contrast media. The decision to proceed with angiography scans in cases of severe calcification was made on a case-by-case basis, without a specific threshold. In younger patients without relevant cardiovascular risk factors (and no expected relevant coronary calcifications), we decided individually not to perform non-contrast-enhanced scans to reduce X-ray exposure.

The CCTA employed either a prospectively ECG-triggered high-speed spiral ("Turbo Flash") or sequential ("Step and Shoot") scan in a cranio-caudal direction, using iodinated contrast media (Imeron 350, Bracco, Konstanz, Germany) at 40 ml for "Turbo Flash" and 50 ml for "Step and Shoot," injected at 5 ml/s. The region of interest (ROI) was the ascending aorta, tracked using bolus techniques. Choice between scan modes depended on heart rate, rhythm, coronary calcification extent, and body weight. Severe motion artifacts post-"Turbo Flash" prompted repeat scans with "Step and Shoot". Patients received sublingual nitroglycerin (0.8 mg) for coronary dilation, with beta blockers (atenolol p.o. or metoprolol i.v.) for heart rates > 65/min, barring contraindications.

### CCTA imaging analysis

We utilized dedicated post-processing workstations (syngo.via, Siemens Healthineers, Forchheim, Germany) for standard image analysis. Quantitative measurement and characterization of coronary plaque burden were conducted using semi-automated software (syngo.via Frontier CT Coronary Plaque Analysis, version 5.0.2, Siemens Healthineers, Forchheim, Germany), which has been previously validated and described in detail [14, 15]. Plaque analysis was supervised by two experienced readers (A.S. and P.B.), both certified with the highest degree in Cardiac Computed Tomography from the German Cardiac Society.

Initially, non-contrast enhanced scans were assessed to calculate the Agatston score. Subsequently, angiography images were analyzed to evaluate image quality, assessability, and coronary dominance. Plaque analysis encompassed all coronary arteries collectively and each vessel individually, evaluating maximum diameter stenosis (in %) as well as plaque volume (in mm^3^) and burden (in %). Obstructive CAD was defined as presence of stenoses with lumen narrowing > 50%, high-grade coronary stenosis were defined as lumen narrowing > 80%. Calcified and non-calcified (including fibrotic and lipid-rich) plaque components were differentiated depending on attenuation values in Hounsfield Units (HU). Calcification was defined as > 190 HU, lipid-rich as < 30 HU, and fibrotic in between. Additionally, high-risk plaque features were defined as a positive remodeling index > 1.1, napkin ring sign, spotty calcifications, LAP (lipid-rich components within fibrotic plaque components), and high-grade stenoses [8].

### Statistical analysis

Statistical analyses were conducted using R (version 4.3.2) and R Core Team (2023), (R Foundation for Statistical Computing, Vienna, Austria). Categorical data are presented as frequencies or percentages, while continuous variables are reported as mean ± standard deviation (SD) or median with IQR. For comparison of continuous variables with normal distribution, student’s t test was employed. Non normally distributed and categorical data were compared by using Fisher’s exact or Kruskal Wallis testing. Correlations were analyzed with Spearman’s rank correlation coefficient. To account for potential confounding factors, a multiple linear regression analysis was performed including age, sex, and low-density lipoprotein (LDL) cholesterol values as covariates. This allowed adjustment for demographic and lipid-related variables, particularly given the known age difference between female and male patients. To account for the cholesterol content of Lp(a), adjusted LDL-cholesterol (LDL-C) levels were calculated under the assumption that approximately 30% of the Lp(a) mass consists of cholesterol. Lp(a) concentrations were initially measured in nmol/L and converted to mg/dL using a standard conversion factor (Lp(a) [mg/dL] ≈ Lp(a) [nmol/L]/2.4). The Lp(a)-adjusted LDL-C was computed using the following formula:

$$\begin{array}{c}\mathrm L\mathrm D\mathrm L-\mathrm C\;\mathrm{adjusted}\;=\;\mathrm L\mathrm D\mathrm L-\mathrm C\;\mathrm{measured}\;-\;0.3\end{array}\times\;\mathrm{Lp}(\mathrm a)\mathrm{mg}/\mathrm{dL}$$

To evaluate potential gender-specific differences, an interaction term was included in the model. The interaction effect was analyzed using the corresponding *p*-value (P_int_).

Statistical significance was assumed with a p-value < 0.05. Our analysis was done to test the hypothesis that elevated Lp(a) levels are associated with increased total plaque volumes and higher rates of high-risk coronary plaque features.

## Results

Complete data were available for 1,946 patients (mean age 63.3 ± 11.1 years, 62.6% male). The primary symptoms indicating the need for CCTA were typical angina pectoris (43.4%) and dyspnea (29.3%). The median Lp(a) value was 19.0 nmol/L (IQR 7.0–72.0 nmol/L), with 336 patients (17.3%) classified as having high Lp(a) levels. The main baseline characteristics and demographics are summarized in Table 1 and Supplementary Table 1, with all laboratory values detailed in Supplementary Tables 2 and 3.

**Table 1:** Baseline characteristics and demographics of the entire study cohort, as well as male and female subgroups

Characteristics	All patients (*N* = 1946)	Male (*N* = 1219)	Female (*N* = 727)	*P*-Value ‡
Demographic				
Age — yr	63.3 ± 11.1	62.0 ± 10.9	65.4 ± 10.9	< 0.001
Male sex — no. (%)	1219 (62.6)			< 0.001
Median body-mass index (IQR)*	26.6 ± 4.5	27.3 ± 4.2	25.4 ± 4.8	< 0.001
Cardiovascular risk factors — no. (%)				
Hypertension	1198 (61.6)	760 (62.3)	438 (60.2)	0.365
Dyslipidemia	962 (49.4)	595 (48.8)	367 (50.5)	0.530
Diabetes mellitus	197 (10.1)	137 (11.2)	60 (8.3)	0.039
Family predisposition	731 (37.6)	441 (36.2)	290 (39.9)	0.123
Nicotine abuse	243 (12.5)	173 (14.2)	70 (9.6)	0.009
Symptoms as CCTA indication — no. (%) †				
Angina pectoris	844 (43.4)	486 (39.9)	358 (49.2)	< 0.001
Dyspnea	571 (29.3)	296 (24.3)	275 (37.8)	< 0.001
Fatigue	222 (11.4)	137 (11.2)	85 (11.7)	0.903
Palpitations	267 (13.7)	152 (12.5)	115 (15.8)	0.059
Exercise-dependent ectopic beats	76 (3.9)	55 (4.5)	21 (2.9)	0.074
Dizziness	62 (3.2)	33 (2.7)	29 (4.0)	0.170
Syncope	36 (1.8)	21 (1.7)	15 (2.1)	0.746
Laboratory values				
GFR — ml/min/1.73m^2^	81.5 ± 16.0	82.4 ± 15.8	79.7 ± 16.2	0.003
NT-proBNP — pg/mL (IQR)	85.0 (38.0–192.0)	64.0 (28.0–168.0)	122.0 (63.0–248.0)	< 0.001
High-sensitivity Troponin T — pg/mL (IQR)	7.0 (5.0–10.0)	8.0 (5.0–12.0)	5.0 (4.0–8.0)	< 0.001
Lipoprotein(a) — nmol/L	57.7 ± 80.0	54.9 ± 76.5	62.2 ± 85.5	0.061
Medical history — no./total no. (%)				
Anticoagulation	247 (12.7)	154 (12.6)	93 (12.8)	0.975
ASS	329 (16.9)	224 (18.4)	105 (14.4)	0.028
ACE inhibitors	273 (14.0)	175 (14.4)	98 (13.5)	0.608
ARBs	554 (28.5)	354 (29.0)	200 (27.5)	0.467
ARNI	21 (1.1)	12 (1.0)	9 (1.2)	0.778
Beta-Blockers	511 (26.3)	272 (22.3)	239 (32.9)	< 0.001
MRA	38 (2.0)	26 (2.1)	12 (1.7)	0.556
SGLT2 inhibitors	2 (0.1)	1 (0.1)	1 (0.1)	1
Statins	420 (21.6)	289 (23.7)	131 (18.0)	0.003
Ezetimib	39 (2.0)	32 (2.6)	7 (1.0)	0.082

Plus–minus values are means ± SD. For continuous variables, the median and interquartile range are presented for non-normally distributed variables. *ACE* inhibitors denotes angiotensin-converting-enzyme inhibitors, *ARBs* angiotensin II receptor blockers, *ARNI* angiotensin receptor-neprilysin inhibitor, *COPD* chronic obstructive pulmonary disease, *CCTA* coronary computed tomography angiography, *GFR* glomerular filtration rate, *IQR* interquartile range, *MRA* mineralocorticoid receptor antagonist, and *SGLT2i* Sodium glucose co-transport 2 inhibitors

* The body-mass index is the weight in kilograms divided by the square of the height in meters

† Some patients with more than one symptom

‡ The *P*-Value refers to the subgroup comparison male/female

### General CCTA findings

We ruled out CCTA-defined coronary atherosclerosis in 629 patients (32.3%) and observed non-obstructive CAD in 981 patients (50.4%), and obstructive CAD in 336 (17.3%). The median total plaque volume per patient was 47.9 mm^3^ (IQR 0.0–170.8 mm^3^), with a median calcified plaque volume of 26.8 mm^3^ (IQR 0.0–104.4 mm^3^), fibrotic plaque volume of 7.5 mm^3^ (IQR 0.0–44.1 mm^3^), and lipid-rich (LAP) volume of 0 mm^3^ (IQR 0.0–1.1 mm^3^). High-risk plaque features were identified in 1090 patients (56%), with 408 patients (21%) exhibiting two or more of these features. Positive remodeling (*n* = 1043, 53.6%), LAP (*n* = 404, 20.8%), and high-grade stenoses (*n* = 104, 5.3%) were the most prevalent high-risk plaque characteristics. The mean maximum stenosis degree was 44.5 ± 27.5%. Male patients exhibited significantly higher plaque volumes (total, calcified, fibrotic, and lipid-rich), more high-risk plaque features (numbers of high-risk features per patient, positive remodeling index, LAP, and high-grade stenoses), and a higher maximum stenosis degree. Table 2 summarizes all results of the coronary plaque analysis. Supplementary Table 4 presents the results of the plaque analysis in patients not receiving statin therapy.

**Table 2:** CCTA findings of the entire study cohort, as well as male and female subgroups

Characteristics	All patients (*N* = 1946)	Male (*N* = 1219)	Female (*N* = 727)	*P*-Value †
General CCTA findings				< 0.001
Normal coronary findings	629 (32.3)	288 (23.6)	341 (46.9)	
Non-obstructive CAD	981 (50.4)	678 (55.6)	303 (41.7)	
Obstructive CAD	336 (17.3)	253 (20.8)	83 (11.4)	
Agatston score (IQR)	30.0 (0.0–194.0)	59.0 (1.4–260.0)	3.0 (0.0–80.3)	< 0.001
Plaque volume — mm3 (IQR)				
Total	47.9 (0.0–170.8)	87.9 (12.4–235.4)	9.8 (0.0–65.0)	< 0.001
Calcified	26.8 (0.0–104.4)	48.6 (5.3–143.1)	6.1 (0.0–40.3)	< 0.001
Fibrotic	7.5 (0.0–44.1)	19.2 (0.6–70.4)	0.2 (0.0–11.9)	< 0.001
Lipid-rich	0.0 (0.0–1.1)	0.1 (0.0–1.9)	0.0 (0.0–0.1)	< 0.001
High-risk plaque features — no. (%)*				
Positive remodeling index > 1.1	1043 (53.6)	759 (62.3)	284 (39.1)	< 0.001
Napkin ring sign	9 (0.5)	7 (0.6)	2 (0.3)	0.551
Spotty calcifications	17 (0.9)	16 (1.3)	1 (0.1)	0.015
Low attenuation plaques	404 (20.8)	319 (26.2)	85 (11.7)	< 0.001
High-grade stenoses	104 (5.3)	82 (6.7)	22 (3.0)	0.001
Patients with the following numbers of high-risk features — no. (%)				< 0.001
0	856 (44.0)	425 (34.9)	431 (59.3)	
1	682 (35.0)	471 (38.6)	211 (29.0)	
≥ 2	408 (21.0)	323 (26.5)	85 (11.7)	
Maximum stenosis degree — %	44.5 ± 27.5	47.4 ± 26.8	38.3 ± 27.9	< 0.001

Plus–minus values are means ± SD. For continuous variables, the median and interquartile range are presented for non-normally distributed variables. *CAD* denotes coronary artery disease, *CCTA* coronary computed tomography angiography, and *IQR* interquartile range

* Some patients with more than one high-risk plaque feature

† The *P*-Value refers to the subgroup comparison male/female

### Lp(a) and its impact on stenosis degree and coronary plaque burden

**Fig. 1:**
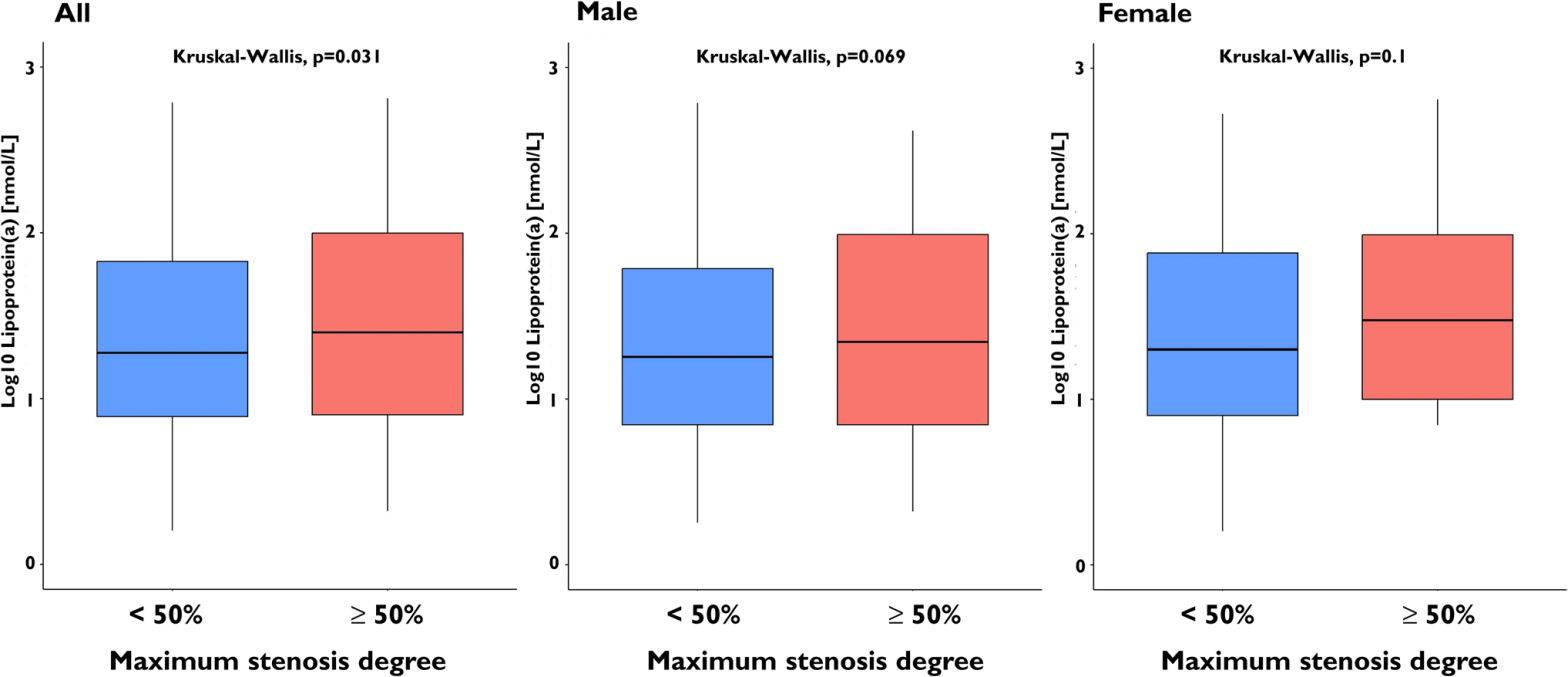
Box plots illustrating the distribution of Lp(a) values in patients with and without obstructive coronary stenosis

**Fig. 2:**
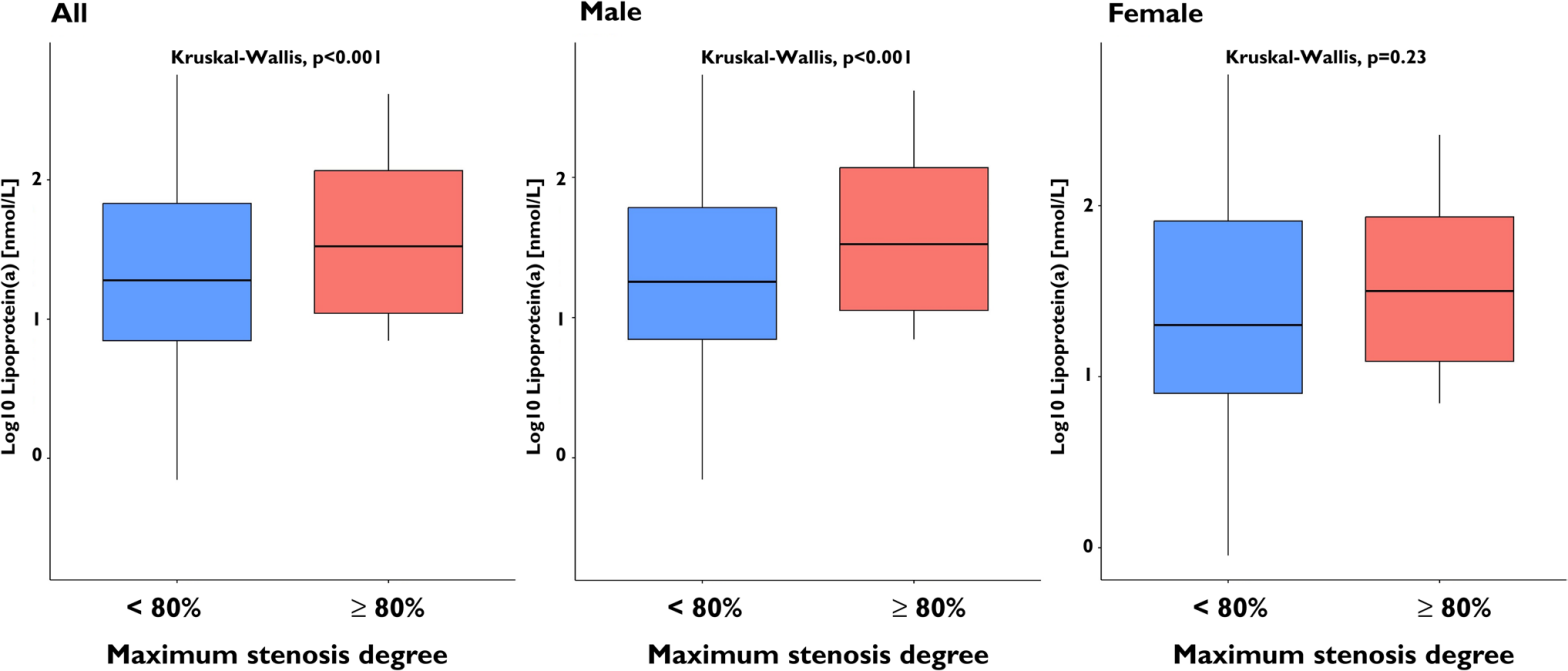
Box plots illustrating the distribution of Lp(a) values and the occurrence of high-grade coronary stenosis

Patients with high Lp(a) levels (≥ 125 nmol/L) had a significantly greater maximum degree of stenosis (49.5 ± 26.4% vs. 43.5 ± 27.6%, *P* = 0.002). The elevated maximum stenosis degree observed in patients with high Lp(a) levels was driven by the male population (53.8 ± 26.0% vs. 46.2 ± 26.8%, *P* = 0.001). Additionally, a significant correlation between Lp(a) values and the frequency of obstructive coronary stenoses was detected in the entire study cohort. This association persisted when LDL cholesterol values were adjusted for the estimated cholesterol content attributable to Lp(a) (odds ratio [OR] for high Lp(a) levels: 1.003; 95% confidence interval [CI]: 1.001 to 1.006; *P* = 0.015). The strongest correlation was observed between Lp(a) levels and high-grade coronary stenoses, identified as a high-risk plaque feature, in both the overall study population and the male subgroup (OR: 1.645; 95% CI: 1.011 to 2.593; *P* = 0.037) (Figs. 1 and 2). This observation was also confirmed in patients not receiving statin therapy (Supplementary Figs. 1 and 2). Female patients exhibited a lower likelihood of developing high-grade coronary stenoses (OR: 0.422; 95% CI: 0.255 to 0.671; *P* < 0.001). However, the sex-related interaction between high Lp(a) levels and high-grade coronary stenoses was not statistically significant (Pint = 0.479).

Although high Lp(a) levels appeared to be associated with higher plaque volumes, these differences were only observed as a trend and were not statistically significant across the entire study cohort. However, in males, high Lp(a) levels were associated with greater total plaque volumes (118.1 [IQR 18.3–284.4] vs. 83.2 [IQR 11.8–226.3] mm^3^, *P* = 0.018, Pint = 0.09), including fibrotic components (27.6 [IQR 2.1–109.9] vs. 18.2 [IQR 0.4–65.0] mm^3^, *P* = 0.011, Pint = 0.013). Of note, Agatston score values were not increased in males with high Lp(a), and calcified plaque volumes differed only marginally (64.0 [IQR 4.0–282.0] vs. 59.0 [IQR 1.0–258.5], *P* = 0.251; and 63.1 [10.0–158.5] vs. 45.2 [4.6–139.0], *P* = 0.096; Pint = 0.479). Table 3 summarizes all CCTA findings for the subgroups categorized by non-high and high Lp(a) levels.

**Table 3:** CCTA findings in correlation of Lp(a) levels for the entire study cohort, as well as male and female subgroups

	All patients (*N* = 1946)			Male (*N* = 1219)			Female (*N *= 727)		
	*Non-high Lp(a)* *(N* = *1610)*	*High Lp(a)* *(N* = *336)*	*P-Value*	*Non-high Lp(a)* *(N* = *1027)*	*High Lp(a)* *(N* = *192)*	*P-Value*	*Non-high Lp(a)* *(N* = *583)*	*High Lp(a)* *(N* = *144)*	*P-Value*
General CCTA findings			0.099			0.059			0.274
Normal coronary findings	530 (32.9)	99 (29.5)		248 (24.1)	40 (20.8)		282 (48.4)	59 (41.0)	
Non-obstructive CAD	815 (50.6)	166 (49.4)		578 (56.3)	100 (52.1)		237 (40.7)	66 (45.8)	
Obstructive CAD	265 (16.5)	71 (21.1)	0.039	201 (19.6)	52 (27.1)	0.018	64 (11.0)	19 (13.2)	0.45
Agatston score (IQR)	29.0 (0.0–186.0)	35.0 (0.0–211.0)	0.33	59.0 (1.0–258.5)	64.0 (4.0–282.0)	0.251	2.0 (0.0–76.0)	8.0 (0.0–125.0)	0.233
Plaque volume — mm^3^ (IQR)									
Total	44.4 (0.0–163.6)	61.9 (3.2–188.8)	0.053	83.2 (11.8–226.3)	118.1 (18.3–284.4)	0.018	8.1 (0.0–61.7)	19.2 (0.0–81.8)	0.061
Calcified	25.8 (0.0–101.9)	32.1 (0.0–112.2)	0.158	45.2 (4.6–139.0)	63.1 (10.0–158.5)	0.096	4.9 (0.0–37.9)	13.1 (0.0–56.9)	0.158
Fibrotic	7.2 (0.0–42.9)	9.3 (0.0–56.4)	0.063	18.2 (0.4–65.0)	27.6 (2.1–109.9)	0.011	0.0 (0.0–11.6)	0.5 (0.0–13.3)	0.114
Lipid-rich	0.0 (0.0–1.0)	0.0 (0.0–1.3)	0.22	0.1 (0.0–1.7)	0.2 (0.0–2.5)	0.229	0.0 (0.0–0.1)	0.0 (0.0–0.4)	0.107
High-risk plaque features — no. (%)*									
Positive remodeling index > 1.1	850 (52.8)	193 (57.4)	0.135	632 (61.5)	127 (66.1)	0.259	218 (37.4)	66 (45.8)	0.078
Napkin ring sign	8 (0.5)	1 (0.3)	0.962	6 (0.6)	1 (0.5)	1	2 (0.3)	0 (0.0)	1
Spotty calcifications	15 (0.9)	2 (0.6)	0.779	14 (1.4)	2 (1.0)	0.989	1 (0.2)	0 (0.0)	1
Low attenuation plaques	327 (20.3)	77 (22.9)	0.319	262 (25.5)	57 (29.7)	0.263	65 (11.1)	20 (13.9)	0.44
High-grade stenoses	79 (4.9)	25 (7.4)	0.081	62 (6.0)	20 (10.4)	0.039	17 (2.9)	5 (3.5)	0.938
Patients with the following numbers of high-risk features — no. (%)			0.192			0.227			0.192
0	721 (44.8)	135 (40.2)		367 (35.7)	58 (30.2)		354 (60.7)	77 (53.5)	
1	562 (34.9)	120 (35.7)		396 (38.6)	75 (39.1)		166 (28.5)	45 (31.2)	
≥ 2	327 (20.3)	81 (24.1)		264 (25.7)	59 (30.7)		63 (10.8)	22 (15.3)	
Maximum stenosis degree — %	43.5 ± 27.6	49.5 ± 26.4	0.002	46.2 ± 26.8	53.8 ± 26.0	0.001	37.2 ± 28.4	42.2 ± 25.5	0.125

Plus–minus values are means ± SD. For continuous variables, the median and interquartile range are presented for non-normally distributed variables. *CAD* denotes coronary artery disease, *CCTA* coronary computed tomography angiography, *IQR* interquartile range, and *Lp(a)* lipoprotein(a)

* Some patients with more than one high-risk plaque feature

**Fig. 3:**
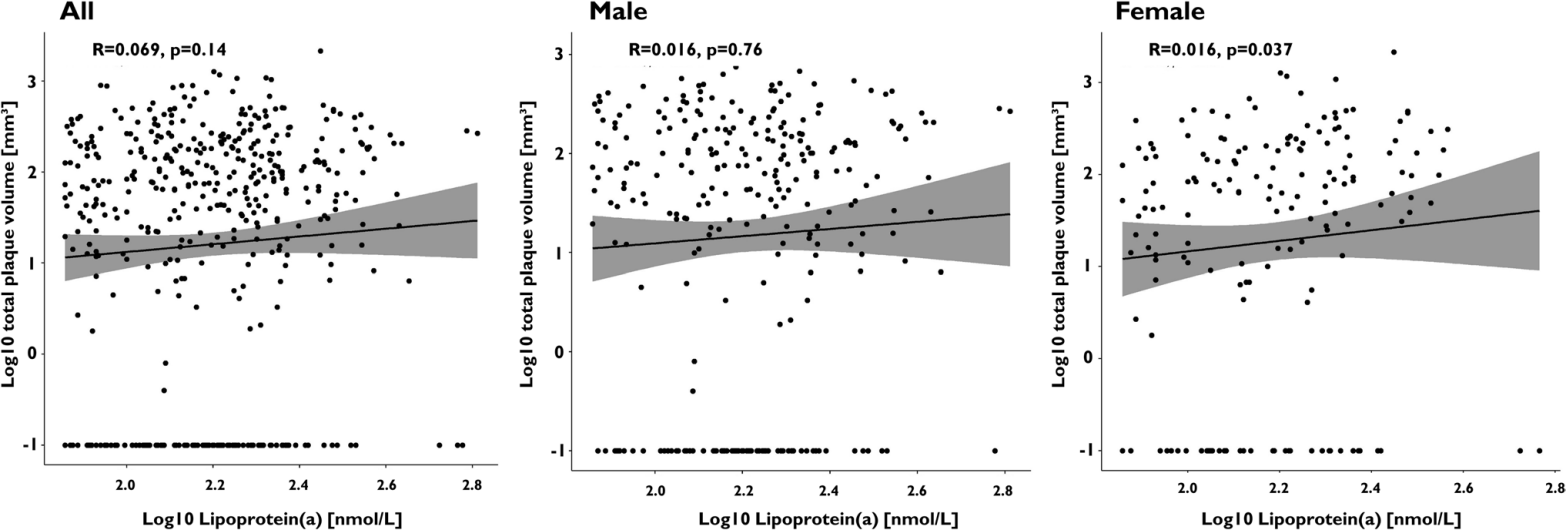
Spearman’s rank correlation coefficients for the analysis of total plaque volume in the overall population, as well as in male and female subgroups

**Fig. 4:**
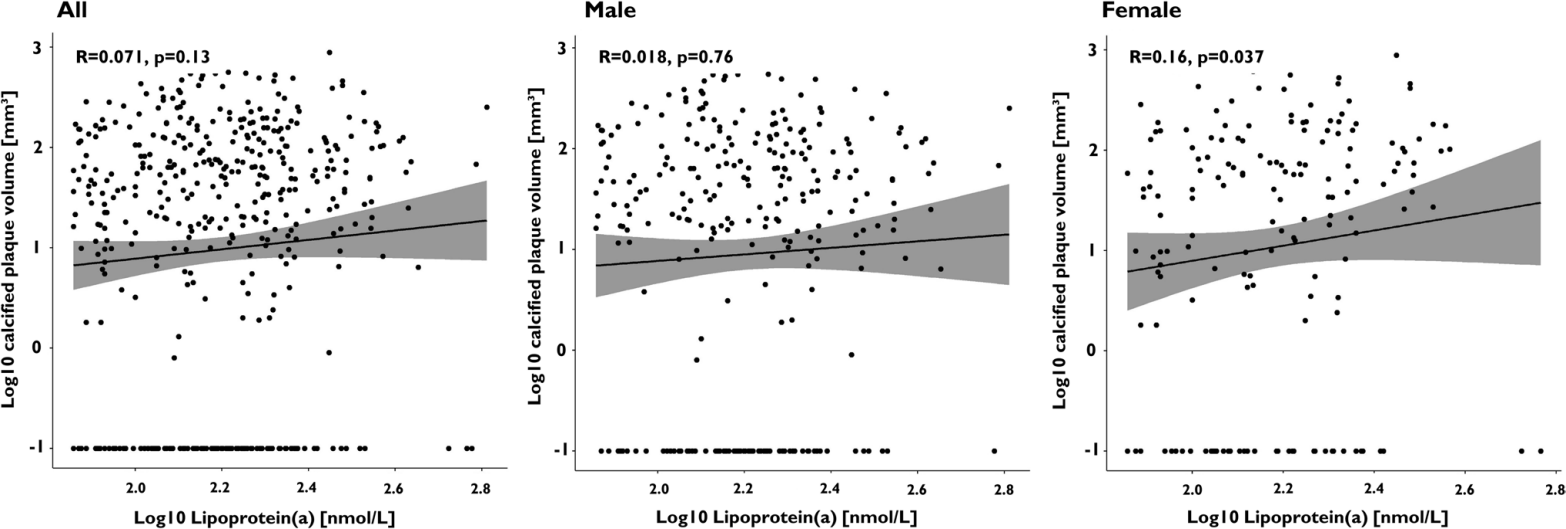
Spearman’s rank correlation coefficients for the analysis of calcified plaque volume in the overall population, as well as in male and female subgroups

**Fig. 5:**
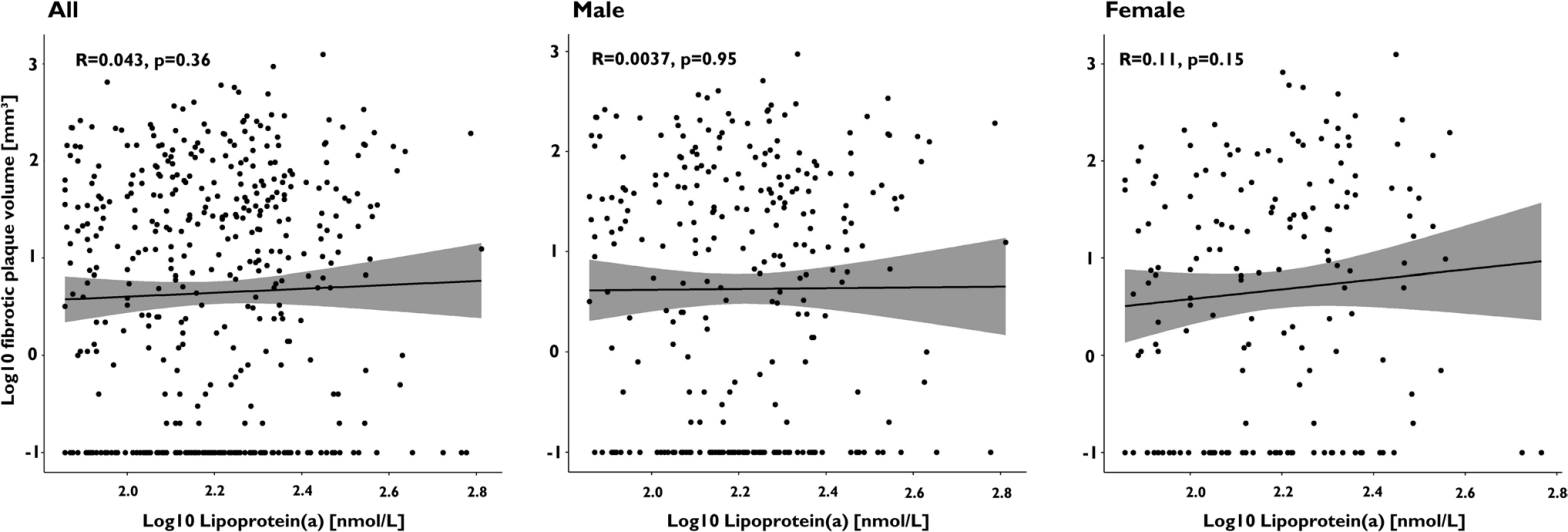
Spearman’s rank correlation coefficients for the analysis of fibrotic plaque volume in the overall population, as well as in male and female subgroups

**Fig. 6:**
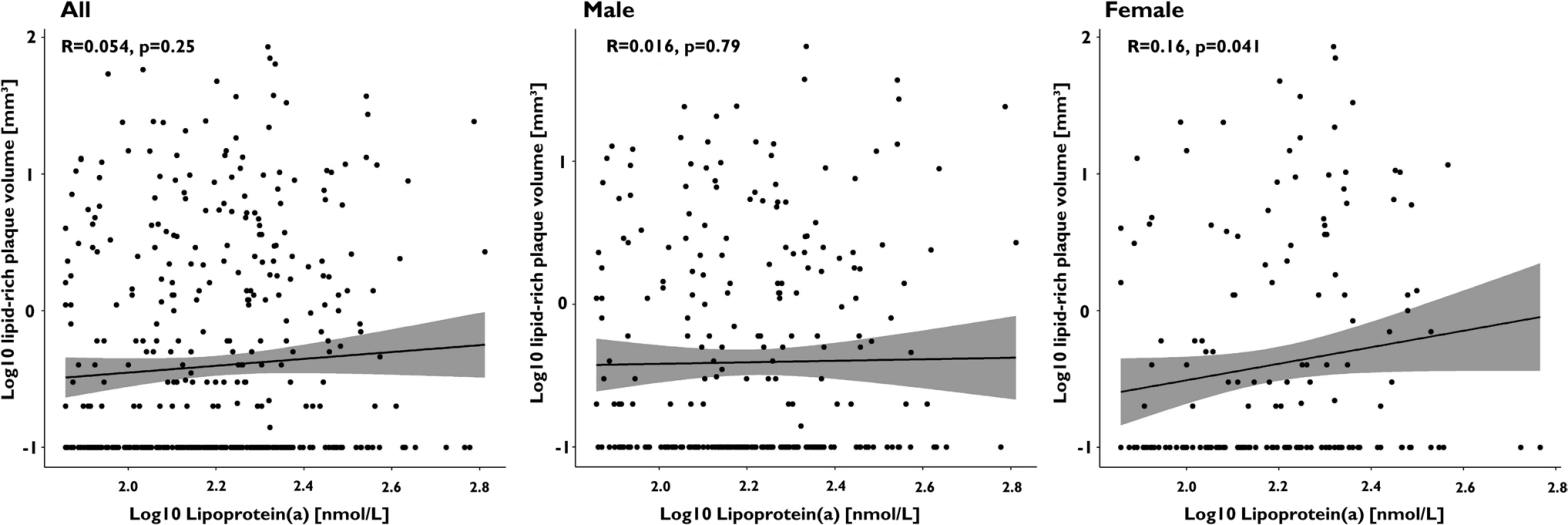
Spearman’s rank correlation coefficients for the analysis of lipid-rich plaque volume in the overall population, as well as in male and female subgroups

Spearman’s rank correlation revealed only a very weak linear correlation between Lp(a) values and total plaque volume, as well as calcified and lipid-rich plaque components in women (R = 0.16, *P* = 0.037; R = 0.16, *P* = 0.037; R = 0.16, *P* = 0.041; Figs. 3, 4, 5, and 6).

Multiple regression analysis, incorporating Lp(a), sex, age, and LDL-cholesterol, was conducted to assess Lp(a)'s impact on plaque burden and coronary stenoses. Lp(a) was confirmed as an independent predictor of total plaque volume, including fibrotic plaque components, and maximum stenosis degree (R = 0.30, *P* = 0.00234; R = 0.23, *P* = 0.00115; R = 0.18, *P* = 0.00234).

## Discussion

This study is the first to provide comprehensive data on the correlation between Lp(a) levels and the extent, severity, and composition of coronary plaque burden in a large outpatient cohort undergoing CCTA for suspected CAD. Our main findings support the hypothesis that Lp(a) levels influence coronary plaque characteristics. Additionally, sex-specific differences were observed, suggesting a potential role in modulating these relationships. Our main findings are:

1. **Coronary Stenoses:** Predominantly driven by the male population, patients with high Lp(a) levels exhibit a significantly greater maximum degree of stenosis. Additionally, a significant correlation was observed between elevated Lp(a) values and the incidence of obstructive coronary stenoses (> 50%) in the entire cohort. Within the male subgroup, there is a trend towards a correlation between high Lp(a) levels and the occurrence of high-risk plaque features, namely high-grade (> 80%) coronary stenoses, without significant gender-related interaction.
2. **Plaque Burden:** High Lp(a) levels ≥ 125 nmol/L are significantly linked to greater fibrotic plaque volumes in men. Additionally, a gender-specific trend towards increased total plaque volume was observed in this group. In women, however, there was only a tendency toward a linear correlation between Lp(a) levels and total plaque volume, including both calcified and lipid-rich components.

Despite widespread scientific agreement on the causal role of Lp(a) in atherosclerotic cardiovascular disease and its potential as a target for intervention, Lp(a) levels are still rarely measured in individuals at high risk. A large international study involving 48,135 patients with known cardiovascular disease from 48 countries found that Lp(a) levels were recorded in fewer than 14% of cases [16]. This is significant because up to 15% of people who experience a first myocardial infarction have no conventional risk factors. Lp(a) could explain why some individuals with few or no traditional risk factors still develop atherosclerotic cardiovascular disease [5]. It is estimated, that 16% to 25% of the general population have genetically determined high Lp(a) levels [12, 16, 17]. Consistent with previous data, 17.3% of patients had high Lp(a) levels in our cohort.

Recent data from the UK Biobank suggest that the risk associated with Lp(a) increases progressively with its concentration, challenging the conventional idea of a specific threshold for atherosclerosis risk. This trend is observed even at lower Lp(a) levels than previously reported and is consistent across various ethnic groups [2]. These findings indicate that the clinical benefit of reducing Lp(a) levels may correlate directly with the extent of the reduction [18]. On the other hand, Ridker et al. reported data from 30,000 initially healthy U.S. women, demonstrating that cardiovascular risk was significantly elevated for those with lipoprotein(a) levels in the highest quintile (quintile 5) [19]. Given some evidence suggesting that even modest reductions in Lp(a) levels may be sufficient to significantly lower the risk of major adverse cardiovascular events (MACE), and the ongoing concerns regarding the potential risks associated with Lp(a)-targeted therapies, the debate around aggressive Lp(a) lowering remains critical and unresolved [5, 20].

Nonetheless, the European Atherosclerosis Society consensus statement acknowledges that, although the relationship between Lp(a) and cardiovascular outcomes is linear, threshold levels in clinical practice help identify Lp(a) values likely to be associated with a significant increase in risk. They advocate for assay-specific cut-offs, similar to those used in cardiac troponin assays, and recommend a pragmatic approach with Lp(a) cut-offs to 'rule out' (< 75 nmol/L) or 'rule in' (≥ 125 nmol/L) risk [5]. The 125 nmol/L threshold has been used in another recent major clinical trial to distinguish between high and non-high Lp(a) levels [12], which is why we also adopted this cut-off in our study.

In the ongoing debate on whether Lp(a) levels correlate linearly with the extent of CAD, or whether a defined cut-off value is more appropriate for risk stratification, our findings provide a novel insight: a sex-specific tendency suggesting that Lp(a) levels may be associated with specific aspects of plaque burden and high-risk features typically regarded as hallmarks of CAD.

In women, however, there was only a tendency toward a correlation between Lp(a) values and total plaque volume, including both calcified and lipid-rich plaque components. In contrast, in male patients, only Lp(a) values above the treshold of ≥ 125 nmol/L were linked to greater fibrotic plaque volumes. Additionally, a gender-specific trend towards increased total plaque volume was observed in this group. As women in our cohort were significantly older than men, all analyses were adjusted for age using multivariable regression models. This approach ensured that the observed sex-specific tendencies were not merely driven by age-related differences, but reflect independent associations.

Regarding the degree of maximum stenosis and the frequency of high-grade coronary stenoses, we detected a correlation with Lp(a) values, both linearly and when using a cut-off, in the entire population. Within the male subgroup, there is a trend towards a correlation between high Lp(a)-values and the occurrence of such high-grade coronary stenoses, without significant gender-related interaction. High-grade stenoses are one of the high-risk plaque features and have been termed a ‘conditio sine qua non’ for the advent of clinical events[21]. In the vast majority of cases, they are assumed to be a prerequisite for atherosclerosis-associated events to occur [21–23]. Against this background, the current data indicate a correlation of elevated Lp(a) with CCTA-markers of high-risk plaque features especially in male patients.

Previous studies have long demonstrated a significant association between high Lp(a) levels and atherosclerosis as detected by CCTA [6, 7]. This association is further supported by a post hoc analysis of six randomized trials using intravascular ultrasound, which showed that elevated Lp(a) was independently associated with increased atheroma volume [24].

The recently published Miami Heart Study, which included 1,785 asymptomatic adults aged 40 to 65 and used the same Lp(a) cut-off for high levels as our study, was the first to evaluate high-risk plaque features. This study found that elevated circulating Lp(a) levels were independently associated with the presence of any coronary plaque and with the presence of ≥ 2 high-risk plaque features [12]. Another trial demonstrated that elevated Lp(a) significantly increased the risk of acute myocardial infarction over an 8-year follow-up period, particularly in patients with LAP identified by CCTA [25].

In our study, the fibrotic plaque volume, was significantly higher in male patients. Calcification, whether measured by the Agatston scoring system or by volumetric assessment, was only marginally different. However, high-grade stenoses were clearly associated with increased Lp(a) levels. Further CCTA high-risk plaque features, such as expansive remodeling or volume of LAP, appeared to demonstrate a weaker association with elevated Lp(a) levels. Compared with previous studies, the varying results regarding non-stenosis related high-risk markers are likely explained by our study population of patients with symptoms suggestive of coronary artery disease instead of asymptomatic individuals. The likelihood of detecting coronary plaque increases with the typicality of the symptoms [26], leading to a higher average plaque volume per patient in our cohort and a lower proportion of patients with normal coronary findings.

Furthermore, we observed higher rates of high-risk features across both non-high and high Lp(a) levels. This can potentially be attributed to the use of a new generation CCTA scanner and advanced quantitative measurement and characterization of coronary plaque burden using artificial intelligence, which were conducted using enhanced semi-automated software.

As mentioned above, Kaiser et al. conducted a study involving 191 patients with stable CAD, assessing plaque burden and LAP features via CCTA over one year. They found no significant differences in plaque volume or composition at baseline between non-high and high Lp(a) levels but observed accelerated progression of LAP as a high-risk feature over this period [10]. In contrast, our study performed CCTAs at a single time point when patients presented with symptoms, requiring us to compare our findings with their baseline CCTA. Our lack of differences in LAP aligns partially with the previous findings; however, discrepancies in plaque volume may be attributed to the fact that Kaiser et al. did not differentiate based on sex, and all included patients had already diagnosed stable CAD [10].

These findings prompt further considerations regarding Lp(a) levels and the occurrence of high-risk plaque features:

1. Are elevated Lp(a) levels primarily responsible for the progression of plaques over time? It has been previously suggested that assessing the association of Lp(a) with high-risk plaque features longitudinally would provide more precise evidence of how Lp(a) contributes to plaque development and progression towards CAD in large cohorts. This could offer critical insights into the true risk for patients with low estimated atherosclerotic cardiovascular disease risk but elevated Lp(a) levels [27].
2. Independently of Lp(a) levels, men showed numerically higher prevalence of LAP and positive remodeling, suggesting a ‘modifying effect‘ of male sex on the correlation between Lp(a) and high-risk coronary plaque features.

The correlation between Lp(a) levels and calcified plaque development remains controversial. The Multi-Ethnic Study of Atherosclerosis (MESA) found that elevated Lp(a) levels were associated with an absolute increase in calcified plaque volume in 5,975 patients who were free of cardiovascular disease at baseline [28]. Similarly, the Atherosclerosis Risk in Communities (ARIC) study demonstrated that high Lp(a) levels in middle age are significantly associated with vascular calcification in older age, reflected by increased CAC, particularly in women [7].

A meta-analysis also identified an independent association between Lp(a) levels and calcified plaque volume [29]. However, other studies have found no significant difference in Agatston scores between individuals with high and normal Lp(a) levels [10, 30]. For instance, the Miami Heart Study found a numerically higher burden of CAC > 0 in those with elevated Lp(a) levels, although these differences were not statistically significant [12].

This debate is further complicated by the use of varying cut-offs for defining high Lp(a) levels, the reliance on Agatston scores to assess calcified plaques, and older CT technologies with various image acquisition protocols and potential for increased inter-scan variability.

In this context, our real-world findings provide a link: Using a state-of-the-art CT scanner in patients with symptoms suggesting CAD, we only detected marginal correlations between Lp(a) values and calcified plaque volume in women and no difference in the Agatston score. In men, no association was found between Lp (a) levels and the Agatston score or calcified plaque volumes.

## Limitations

This registry is limited by its monocentric and retrospective design, which may restrict the generalizability of the findings to broader settings or populations. The retrospective nature of the study introduces potential biases in data collection and analysis. However, these limitations are mitigated by the study's reflection of real-world clinical practice and the consistent use of a single, state-of-the-art imaging scanner over an extended period, thereby enhancing the internal validity of the results. Additionally, the study’s strength lies in its focus on patients with a clear clinical indication for imaging. An additional limitation is the exclusion of CTOs, which were omitted due to the technical challenges in performing a reliable plaque analysis in these lesions. A notable limitation is that CCTA images were obtained at a single time point, which prevents us from predicting the progression of coronary stenosis and plaque development over time in relation to Lp(a) levels. Future research using multicentric approaches and prospective study designs could further validate and expand upon the findings of this investigation.

Moreover, we acknowledge that sex-specific differences in the decision to perform CCTA may have introduced a selection bias. Variations in clinical presentation, pre-test probability assessment, or referral behavior could have led to different inclusion thresholds for male and female patients. As such, the observed sex-related differences may partly reflect these selection effects in addition to biological factors.

A final limitation is that our analysis included only patients who underwent CCTA for clinical indications and had Lp(a) measurements between July 2017 and June 2020. As previously described, Lp(a) measurements were routinely performed in all patients seen in our in-house outpatient cardiology practice as part of cardiovascular risk assessment. Patients without Lp(a) values were typically referred from external cardiology practices. However, these patients represent the same clinical population, and no systematic differences in care pathways are expected, as equivalent cardiologic evaluations were performed across both settings. We therefore consider the analysed cohort to be representative of the broader registry population during the defined study period.

## Conclusion

Our study of a large outpatient cohort undergoing CCTA for suspected CAD reveals novel sex-specific differences between Lp(a) levels and coronary plaque characteristics, suggesting a potential role in modulating these relationships. High Lp(a) levels in men seems to be associated with increased fibrotic plaque volumes and may contribute to greater total plaque burden and the occurrence of high-grade stenoses. In women, however, there was only a tendency toward a linear correlation between Lp(a) values and total plaque volumes was seen.

## Supplementary information

Below is the link to the electronic supplementary material.

ESM1 (TIF 2.13 MB)

ESM2 (TIF 2.01 MB)

ESM3 (DOCX 35.6 KB)

ESM4 (DOCX 35.5 KB)

ESM5 (DOCX 35.0 KB)

ESM6 (DOCX 34.5 KB)

## Data Availability

Data included in this manuscript will be made available upon reasonable requests.

## Abbreviations

CAC: Coronary artery calcium
CAD: Coronary artery disease
CK: Creatine kinase
CCTA: Coronary computed tomography angiography
ECG: Electrocardiogram
hsTnT: high-sensitivity troponin T
LAP: Low attenuation plaques
Lp(a): Lipoprotein(a)
