# Supplementary figures and images for: Sex-specific influence of Lipoprotein(a) levels on coronary plaque characteristics: - The COPRODUCTION Registry -
Source: Clin Res Cardiol. 2025 Oct 9;114(12):1739–51. doi: 10.1007/s00392-025-02770-w (PMC12708768; doi:10.1007/s00392-025-02770-w)

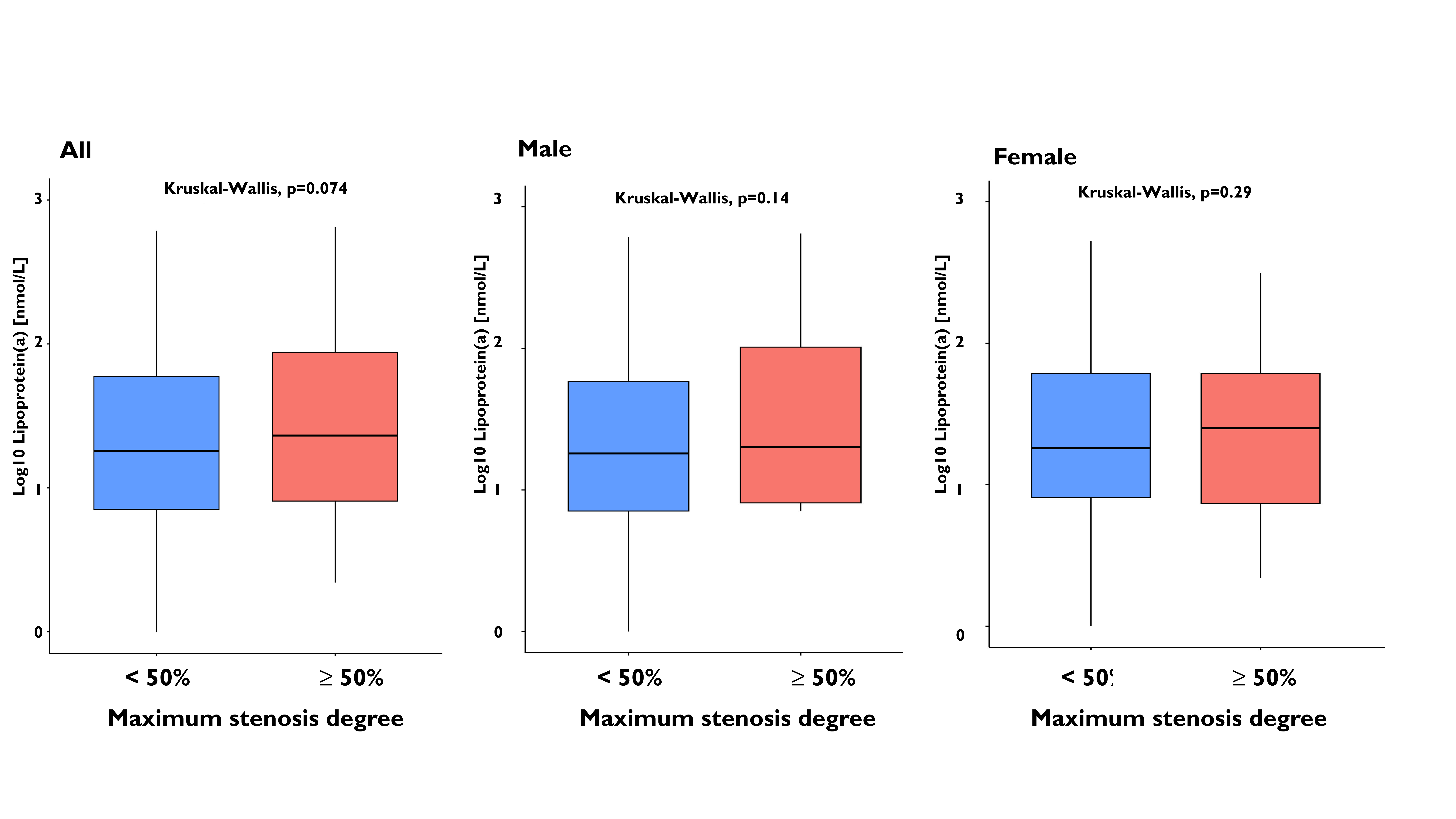

Supplement: Supplementary file 1 — (TIF 2.13 MB) [file 392_2025_2770_MOESM1_ESM.tif]

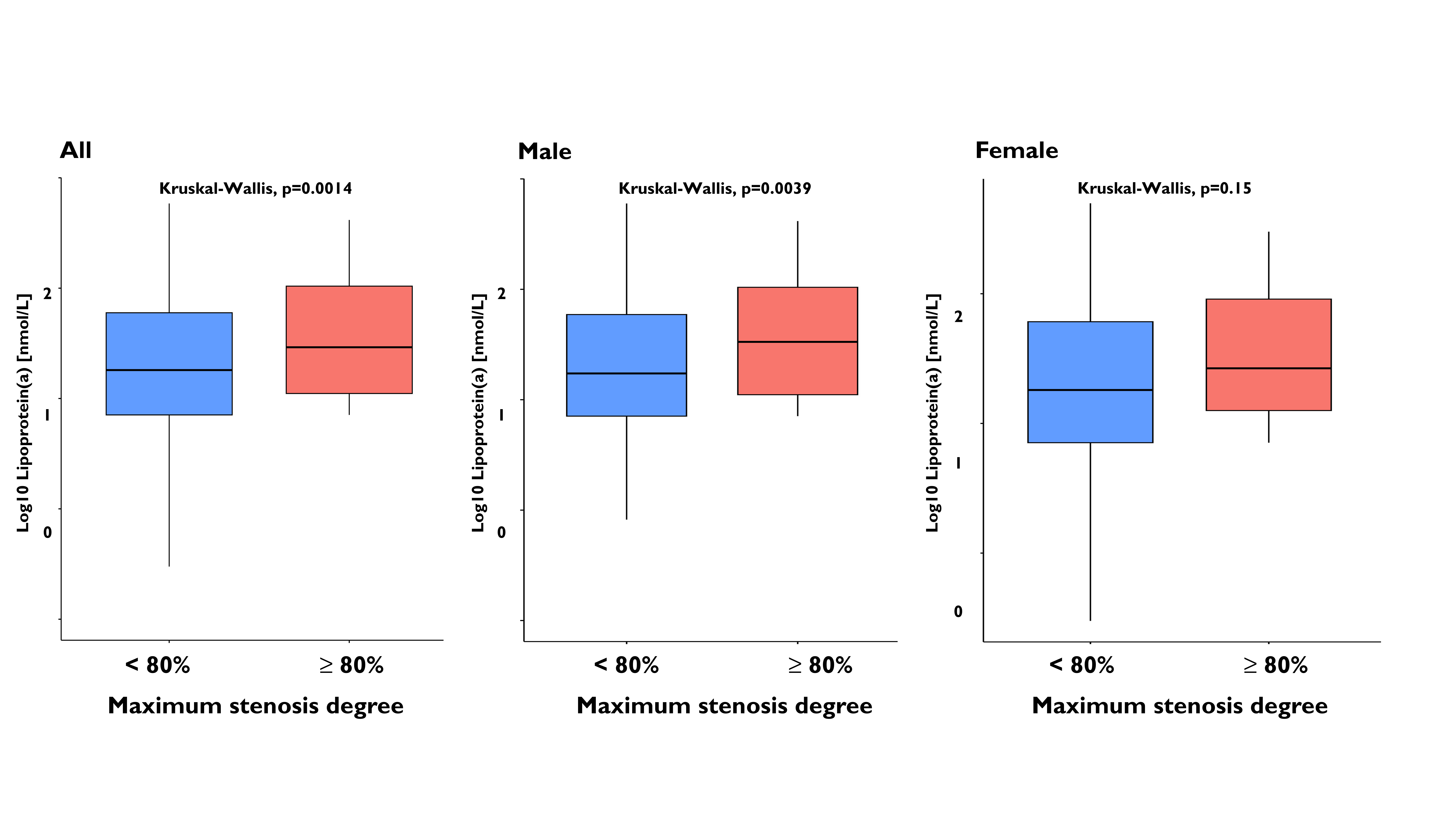

Supplement: Supplementary file 2 — (TIF 2.01 MB) [file 392_2025_2770_MOESM2_ESM.tif]
