# Supplementary material for: Sex-specific influence of Lipoprotein(a) levels on coronary plaque characteristics: - The COPRODUCTION Registry -
Source: Clin Res Cardiol. 2025 Oct 9;114(12):1739–51. doi: 10.1007/s00392-025-02770-w (PMC12708768; doi:10.1007/s00392-025-02770-w)
Supplement: Supplementary file 3 — (DOCX 35.6 KB) [file 392_2025_2770_MOESM3_ESM.docx]

**Supplement Table 1 – Baseline characteristics and demographics in correlation of Lp(a) levels.**

| **Characteristics** | **Non-high Lp(a)**  **(N=1610)** | **High Lp(a)**  **(N=336)** | **P-Value** |
| --- | --- | --- | --- |
| Demographic |  |  |  |
| Age — yr | 63.3±11.1 | 63.2±10.7 | 0.942 |
| Male sex — no. (%) | 1027 (63.8) | 192 (57.1) | 0.026 |
| Median body-mass index (IQR)* | 26.6±4.5 | 26.6±4.2 | 0.873 |
| Cardiovascular risk factors — no. (%) |  |  |  |
| Hypertension | 987 (61.3) | 211 (62.8) | 0.497 |
| Dyslipidemia | 745 (46.3) | 217 (64.6) | <0.001 |
| Diabetes mellitus | 174 (10.8) | 23 (6.8) | 0.041 |
| Family predisposition | 585 (36.3) | 146 (43.5) | 0.016 |
| Nicotine abuse | 211 (13.1) | 32 (9.6) | 0.118 |
| Symptoms as CCTA indication — no. (%)† |  |  |  |
| Angina pectoris | 714 (44.3) | 130 (38.7) | 0.084 |
| Dyspnea | 472 (29.3) | 99 (29.5) | 0.931 |
| Fatigue | 193 (12.0) | 28 (8.3) | 0.105 |
| Palpitations | 221 (13.7) | 46 (13.7) | 1 |
| Exercise-dependent ectopic beats | 64 (4.0) | 12 (3.6) | 0.958 |
| Dizziness | 51 (3.2) | 11 (3.3) | 1 |
| Syncope | 32 (2.0) | 4 (1.2) | 0.455 |
| Medical history — no./total no. (%) |  |  |  |
| Anticoagulation | 211 (13.1) | 36 (10.7) | 0.268 |
| ASS | 264 (16.4) | 65 (19.3) | 0.196 |
| ACE inhibitors | 218 (13.5) | 55 (16.4) | 0.189 |
| ARBs | 461 (28.6) | 93 (27.7) | 0.855 |
| ARNI | 16 (1.0) | 5 (1.5) | 0.601 |
| Beta-Blockers | 424 (26.3) | 87 (25.9) | 0.986 |
| MRA | 34 (2.1) | 4 (1.2) | 0.378 |
| SGLT2 inhibitors | 2 (0.1) | 0 (0.0) | 1 |
| Statins | 326 (20.2) | 94 (28.0) | 0.002 |
| Ezetimib | 27 (1.7) | 12 (3.6) | 0.025 |

Plus–minus values are means ±SD. For continuous variables, the median and interquartile range are presented for non-normally distributed variables. ACE inhibitors denotes angiotensin-converting-enzyme inhibitors, ARBs angiotensin II receptor blockers, ARNI angiotensin receptor-neprilysin inhibitor, COPD chronic obstructive pulmonary disease, CCTA coronary computed tomography angiography, IQR interquartile range, MRA mineralocorticoid receptor antagonist, and SGLT2i Sodium glucose co-transport 2 inhibitors.

* The body-mass index is the weight in kilograms divided by the square of the height in meters.

†Some patients with more than one symptom.
