# Supplementary material for: Sex-specific influence of Lipoprotein(a) levels on coronary plaque characteristics: - The COPRODUCTION Registry -
Source: Clin Res Cardiol. 2025 Oct 9;114(12):1739–51. doi: 10.1007/s00392-025-02770-w (PMC12708768; doi:10.1007/s00392-025-02770-w)
Supplement: Supplementary file 4 — (DOCX 35.5 KB) [file 392_2025_2770_MOESM4_ESM.docx]

| **Characteristics** | **All patients**  **(N= 1946)** | **Male**  **(N=1219)** | **Female**  **(N=727)** | **P-Value *** |
| --- | --- | --- | --- | --- |
| Baseline laboratory values |  |  |  |  |
| Hemoglobin — g/dL | 14.5±1.4 | 15.0±1.2 | 13.6±1.1 | <0.001 |
| Hematocrit — % | 42.5±4.1 | 43.9±3.8 | 4.0±3.3 | <0.001 |
| Erythrocytes — x10^6^/µL | 4.6±0.5 | 4.8±0.5 | 4.4±0.4 | <0.001 |
| Leukocytes — x10^9^/L (IQR) | 6.4 (5.4–7.5) | 6.4 (5.5–7.5) | 6.3 (5.2–7.6) | 0.105 |
| Platelets x10^9^/L | 235.6±59.9 | 223.2±54.5 | 258.0±62.7 | <0.001 |
| INR levels (IQR) | 1.0 (0.9–1.1) | 1.0 (1.0–1.1) | 1.0 (0.9–1.0) | <0.001 |
| PTT — s (IQR) | 29.0 (27.6–31.2) | 29.3 (27.8–31.2) | 28.9 (27.2–31.2) | 0.017 |
| HbA1c — % (IQR) | 5.5 (5.3–5.7) | 5.5 (5.3–5.8) | 5.5 (5.2–5.7) | 0.013 |
| CRP — mg/L (IQR) | 1.2 (0.6–2.6) | 1.2 (0.6–2.5) | 1.3 (0.6–2.8) | 0.474 |
| Ferritin — ng/mL (IQR) | 179.0 (102.0–287.0) | 231.0 (142.0–334.0) | 110.0 (68.5–176.0) | <0.001 |
| Cardiac biomarkers |  |  |  |  |
| NT-proBNP — pg/mL (IQR) | 85.0 (38.0–192.0) | 64.0 (28.0–168.0) | 122.0 (63.0–248.0) | <0.001 |
| hsTnT — pg/mL (IQR) | 7.0 (5.0–10.0) | 8.0 (5.0–12.0) | 5.0 (4.0–8.0) | <0.001 |
| CK — U/L (IQR) | 101.0 (74.0–147.0) | 113.0 (83.8–162.0) | 84.0 (62.0–112.8) | <0.001 |
| CK-MB — U/L (IQR) | 2.4 (1.7–3.7) | 2.6 (1.8–4.0) | 2.2 (1.5–3.2) | <0.001 |
| Lipid laboratory values |  |  |  |  |
| Total cholesterol — mg/dL | 201.6±43.6 | 194.6±43.2 | 214.7±41.5 | <0.001 |
| LDL-C — mg/dL | 130.2±38.9 | 127.7±38.1 | 135.0±39.8 | 0.002 |
| HDL-C — mg/dL | 58.9±16.9) | 53.3±13.6 | 69.2±17.6 | <0.001 |
| Triglycerides —mg/L (IQR) | 123.0 (87.0–185.0) | 132.0 (92.0–196.0) | 110.0 (81.0–159.8) | <0.001 |
| Lipoprotein(a) — nmol/L | 57.7±80.0 | 54.9±76.5 | 62.2±85.5 | 0.061 |
| Renal laboratory values |  |  |  |  |
| Creatinine — mg/dL | 0.9±0.2 | 1.0±0.2 | 0.8±0.2 | <0.001 |
| GFR — ml/min/1.73m2 | 81.5±16.0 | 82.4±15.8 | 79.7±16.2 | 0.003 |
| Uric acid — mg/dL (IQR) | 5.5 (4.6–6.4) | 5.9 (5.1–6.8) | 4.7 (4.0–5.5) | <0.001 |
| Sodium — mmol/L | 139.8±2.3 | 139.9±2.1 | 139.5±2.6 | 0.006 |
| Potassium — mmol/L | 4.3±0.4 | 4.3±0.4 | 4.3±0.4 | 0.012 |
| Liver laboratory values |  |  |  |  |
| AST — U/L (IQR) | 23.0 (19.0–28.0) | 24.0 (20.0–29.0) | 21.0 (18.0–25.0) | <0.001 |
| ALT — U/L (IQR) | 23.0 (17.0–32.0) | 26.0 (19.0–36.0) | 18.0 (15.0–24.8) | <0.001 |
| Total bilirubin — mg/dL (IQR) | 0.5 (0.3–0.7) | 0.5 (0.4–0.7) | 0.4 (0.3–0.6) | 0.049 |

**Supplement Table 2 – Laboratory values of the entire study cohort as well as the male and female subgroups at baseline.**

Plus–minus values are means ±SD. For continuous variables, the median and interquartile range are presented for non-normally distributed variables. ALT denotes alanine aminotransferase, AST aspartate aminotransferase, CK creatine kinase, CRP c-reactive protein, GFR glomerular filtration rate, HbA1c glycated hemoglobin, HDL-C high-density lipoprotein cholesterol, INR international normalized ratio, IQR interquartile range, PTT partial thromboplastin time, LDL low-density lipoprotein cholesterol, Lp(a) lipoprotein(a), and U units.

* The P-Value refers to the subgroup comparison male/female.
