# Supplementary material for: Sex-specific influence of Lipoprotein(a) levels on coronary plaque characteristics: - The COPRODUCTION Registry -
Source: Clin Res Cardiol. 2025 Oct 9;114(12):1739–51. doi: 10.1007/s00392-025-02770-w (PMC12708768; doi:10.1007/s00392-025-02770-w)
Supplement: Supplementary file 5 — (DOCX 35.0 KB) [file 392_2025_2770_MOESM5_ESM.docx]

**Supplement Table 3 – Laboratory values in correlation of Lp(a) levels.**

| **Characteristics** | **Non-high Lp(a)**  **(N=1610)** | **High Lp(a)**  **(N=336)** | **P-Value** |
| --- | --- | --- | --- |
| Baseline laboratory values |  |  |  |
| Hemoglobin — g/dL | 14.5±1.4 | 14.5±1.3 | 0.891 |
| Hematocrit — % | 42.5±4.0 | 42.5±4.2 | 0,925 |
| Erythrocytes — x10^6^/µL | 4.6±0.5 | 4.6±0.5 | 0,553 |
| Leukocytes — x10^9^/L (IQR) | 6.4 (5.5–7.6) | 6.2 (5.4–7.5) | 0.236 |
| Platelets x10^9^/L | 235.1±60.6 | 237.6±56.3 | 0,556 |
| INR levels (IQR) | 1.0 (0.9–1.1) | 1.0 (0.9–1.1) | 0.572 |
| PTT — s (IQR) | 29.1 (27.7–31.2) | 29.3 (27.4–31.3) | 0.960 |
| HbA1c — % (IQR) | 5.5 (5.3–5.8) | 5.5 (5.3–5.7) | 0.096 |
| CRP — mg/L (IQR) | 1.2 (0.6–2.7) | 1.2 (0.6–2.2) | 0.513 |
| Ferritin — ng/mL (IQR) | 175.0 (102.0–285.0) | 191.5 (114.5–297.0) | 0.275 |
| Cardiac biomarkers |  |  |  |
| NT-proBNP — pg/mL (IQR) | 84.0 (38.0–188.0) | 93.0 (40.0–234.0) | 0.607 |
| hsTnT — pg/mL (IQR) | 7.0 (5.0–10.0) | 7.0 (5.0–11.0) | 0.780 |
| CK — U/L (IQR) | 101.0 (74.0–146.0) | 101.0 (73.0–153.0) | 0.812 |
| CK-MB — U/L (IQR) | 2.4 (1.7–3.7) | 2.5 (1.7–4.0) | 0.700 |
| Lipid laboratory values |  |  |  |
| Total cholesterol — mg/dL | 200.6±43.1 | 206.3±46.0 | 0.093 |
| LDL-C — mg/dL | 129.2±38.4 | 135.4±40.7 | 0.038 |
| HDL-C — mg/dL | 58.8±16.9 | 59.1±16.7 | 0.798 |
| Triglycerides —mg/L (IQR) | 124.0 (88.0–185.0) | 117.0 (86.0–178.0) | 0.553 |
| Lipoprotein(a) — nmol/L | 26.5±28.3 | 208.2±78.5 | <0.001 |
| Renal laboratory values |  |  |  |
| Creatinine — mg/dL | 0.9±0.2 | 0.9±0.2 | 0.083 |
| GFR — ml/min/1.73m2 | 81.3±16.0 | 82.6±16.1 | 0.254 |
| Uric acid — mg/dL (IQR) | 5.5 (4.6–6.5) | 5.3 (4.6–6.4) | 0.641 |
| Sodium — mmol/L | 139.8±2.3 | 139.7±2.2 | 0.618 |
| Potassium — mmol/L | 4.3±0.4 | 4.3±0.4 | 0.781 |
| Liver laboratory values |  |  |  |
| AST — U/L (IQR) | 23.0 (19.0–28.0) | 23.0 (19.0–27.0) | 0.443 |
| ALT — U/L (IQR) | 23.0 (17.0–32.0) | 22.0 (17.0–32.8) | 0.982 |
| Total bilirubin — mg/dL (IQR) | 0.5 (0.3–0.7) | 0.6 (0.4–0.6) | 0.743 |

Plus–minus values are means ±SD. For continuous variables, the median and interquartile range are presented for non-normally distributed variables. ALT denotes alanine aminotransferase, AST aspartate aminotransferase, CK creatine kinase, CRP c-reactive protein, GFR glomerular filtration rate, HbA1c glycated hemoglobin, HDL-C high-density lipoprotein cholesterol, INR international normalized ratio, IQR interquartile range, PTT partial thromboplastin time, LDL low-density lipoprotein cholesterol, Lp(a) lipoprotein(a), and U units.
