# Supplementary material for: Sex-specific influence of Lipoprotein(a) levels on coronary plaque characteristics: - The COPRODUCTION Registry -
Source: Clin Res Cardiol. 2025 Oct 9;114(12):1739–51. doi: 10.1007/s00392-025-02770-w (PMC12708768; doi:10.1007/s00392-025-02770-w)
Supplement: Supplementary file 6 — (DOCX 34.5 KB) [file 392_2025_2770_MOESM6_ESM.docx]

**Supplementary Table 4 – CCTA findings in relation to Lp(a) levels for the overall cohort and for male and female subgroups among patients not receiving statin therapy.**

|  | **All patients**  **(N= 1514)** | | |
| --- | --- | --- | --- |
|  | ***Non-high Lp(a)***  ***(N=1276)*** | ***High Lp(a)***  ***(N=238)*** | ***P-Value*** |
| General CCTA findings |  |  | 0.077 |
| Normal coronary findings | 469 (36.8) | 87 (36.6) |  |
| Non-obstructive CAD | 620 (48.6) | 103 (43.3) |  |
| Obstructive CAD | 187 (14.7) | 48 (20.2) |  |
| Agatston score (IQR) | 15.0 (0.0–141.8) | 15.0 (0.0–158.0) | 0.953 |
| Plaque volume — mm³ (IQR) |  |  |  |
| Total | 30.4 (0.0–139.3) | 37.5 (0.0–171.9) | 0.337 |
| Calcified | 16.2 (0.0–81.5) | 17.1 (0.0–97.0) | 0.688 |
| Fibrotic | 4.3 (0.0–35.3) | 5.4 (0.0–54.6) | 0.229 |
| Lipid-rich | 0.0 (0.0–0.9) | 0.0 (0.0–1.1) | 0.717 |
| High-risk plaque features — no. (%)* |  |  |  |
| Positive remodeling index >1.1 | 617 (48.4) | 118 (49.6) | 0.782 |
| Napkin ring sign | 4 (0.3) | 0 (0.0) | 0.859 |
| Spotty calcifications | 9 (0.7) | 0 (0.0) | 0.401 |
| Low attenuation plaques | 246 (19.3) | 52 (21.8) | 0.408 |
| High-grade stenoses | 56 (4.4) | 16 (6.7) | 0.165 |
| Patients with the following numbers of high-risk features — no. (%) |  |  | 0.192 |
| 0 | 721 (44.8) | 135 (40.2) |  |
| 1 | 562 (34.9) | 120 (35.7) |  |
| ≥2 | 327 (20.3) | 81 (24.1) |  |
| Maximum stenosis degree — % | 38.7±30.9 | 46.3±32.0 | 0.034 |

Plus–minus values are means ±SD. For continuous variables, the median and interquartile range are presented for non-normally distributed variables. CAD denotes coronary artery disease, CCTA coronary computed tomography angiography, IQR interquartile range, and Lp(a) lipoprotein(a). * Some patients with more than one high-risk plaque feature.
